# Do Fakes Exist? Trade and Consumption of Sex Enhancers in Harare's Avenues

**DOI:** 10.1080/13696815.2022.2136630

**Published:** 2022-12-08

**Authors:** Ushewedu Kufakurinani

**Affiliations:** a Department of Global Health and Social Medicine, Kings College London, London, UK; b Department of Anthropology and Development Studies, University of Johannesburg, Johannesburg, South Africa; c Department of History, Archaeology and Development Studies, Great Zimbabwe University, Masvingo, Zimbabwe

**Keywords:** Efficacy, sex enhancers, informal trade, fake sex enhancers, economic crisis, masculinity, authenticity, sexual prowess

## Abstract

After the ban of over-the-counter sex enhancers in 2013 by the Medical Control Authority of Zimbabwe (MCAZ), the streets of the inner city area of Harare called The Avenues became a haven for illegal traders in sex enhancers. This article explores how products that are banned by the state acquire their own agencies. From the traders’ perspectives, the point of departure to understanding efficacy is not the sex enhancers, but their clients’ needs and their body systems. Thus, the authenticity of the products ceases to be a point of reference. The trade and consumption of sex enhancers amongst Zimbabweans is itself intricately intertwined with the broader political economy of the country. A deteriorating economy has engendered a crisis of masculinity which has contributed towards increased trade and consumption in sex enhancers. The article argues that drug efficacy is a complex phenomenon, whose conceptualisation and manifestations are fluid and not always pharmacological. The research documents six months in The Avenues as research “field”, enriched by both online and traditional ethnographies. The analysis aims to understand perceptions of and dynamics around the efficacy of sex enhancers sold in Harare’s urban streets.

## A Deteriorating Economy, Masculinity in Crisis and the 2013 Ban of Sex Enhancers

Zimbabwe has been in an economic crisis for over two decades, a crisis which can be traced back to the IMF and World Bank-led Economic Structural Adjustment Programme (ESAP). Between 1992 and 1995 these liberalisation programmes crippled all sectors of the economy, as the government withdrew or reduced its participation. ESAP also caused large-scale labour retrenchments which caused an unprecedented burgeoning of the informal economy (Mlambo 1997). The new millennium has only plunged the country further into crisis as the Fast-Track Land Resettlement Programme^1^ from 2000 derailed the country’s economy and a series of political and economic missteps led to hyperinflation and massive economic crisis (Chiumbu and Musemwa 2012; Kufakurinani 2021). The nation never fully recovered from these challenges and today the economy remains largely informal, as manufacturing industries have failed to revive or take off anew. The economic crisis helps to explain not only the rise of the informal economy in which sex enhancer traders operate, but also to a certain extent the increase in the uptake of sex enhancers by men.

In a conversation with a male colleague from the University of Zimbabwe (UZ) about sex enhancers, he expressed the opinion that, because of the harsh economic environment and the pressures of sustaining families, “most men” in the country were experiencing erectile dysfunction. Zimbabwe is a patriarchal society where masculinities are constructed around men’s ability to provide for families and partners. A female colleague from UZ echoed the same sentiments about the economic crisis and its threat to men’s ability to provide as expected by patriarchy. Zimbabwean men are under so much pressure because of an underperforming economy, she remarked, that they really feel the need to take these sex enhancers. Her opinion, she insisted, was that they should only take natural herbs, as these were “safer”. In scholarship about Ghanaian attitudes to “male sexual dysfunction”, authors have argued that sexual dysfunction devastates men’s ego and annihilates the very essence of masculinity (Amidu et al. 2010; Yiana, Ziblim, and Margaret 2018). Taking sex enhancers is often seen as one way to overcome the crisis of masculinity induced by an unstable and deteriorating economy.

Rosalijin Both, in her study of Ethiopia, also links economic hardships to the uptake of sex enhancers, noting that men who lacked “the educational and employment opportunities to become financial providers, compensate for feelings of disempowerment and unmanliness by exerting a kind of masculinity based on sexual prowess” (2016, 496). This demonstrates how a crisis in one form of masculinity, such as the ability to provide materially, is compensated for by another form of masculinity articulated in the form of sexual prowess. Linked to the issues of sexual prowess are issues of multiple partners and the need to satisfy them. In Zimbabwe, there has emerged what has been called the “small-house” culture. This, in many ways, is a modernised form of polygamy. Lois Chingandu notes that small houses are defined by

many Zimbabweans as; an informal, long term, secret sexual relationship with another woman who is not a man’s legal wife, carried on in a house that is a smaller version of the man’s own home in another residential suburb … . For as long as it is practically possible, the small house is kept secret from the legal wife and her children (Chingandu n.d.).

Sometimes unmarried young men also have multi-partners whom they are under pressure to satisfy. Writing about young men in Ethiopia, Both observes that these young men took Viagra to “quell anxieties about what they perceive as women’s growing expectations of their sexual performance” (2016, 496). These perspectives and assumptions were also expressed by the traders with whom I spoke in The Avenues.

The MCAZ banned the selling of sex enhancers over the counter “owing to their side effects and addictive components” deemed to put many lives at risk (Gwarisa 2017). The MCAZ also expressed concern about “their safety and authenticity”.^2^ Before 2013, sex enhancers were mostly sold over the counter in pharmacies. These came from various parts of the world – China, India and even neighbouring Zambia. In 2013, the MCAZ withdrew the registration of most of these sex enhancers and proceeded to ban their over-the-counter sale claiming that they had been duped into registering dangerous and unsafe drugs. After the ban and following a protracted economic crisis, the streets of The Avenues became a haven for illegal traders in sex enhancers. My analysis is that the ban fuelled the subterranean trade in sex enhancers and with it complex transactions and marketing technologies. The trade in sex enhancers has become part of the informal economy, a term which Kristin Peterson defines in this context of research on drugs trade as connoting “capital, goods, and practices that largely bypass state oversight” (2014, 83). The informal market is a space where official regulatory standards and official standards are not always subscribed to and are, therefore, under suspicion. Using the Nigerian case, Peterson notes how traders in these unofficial markets were “deemed charlatans, fraudsters, fakers and evil doers” (2021, 345). The issue of suspicion relates to the efficacy of medications and prevalence of fake drugs that find their way into the marketplace. Indeed, as Patricia Kingori has argued, “there are few areas of everyday life that have not been clouded in suspicion with questions raised about their authenticity” (2021, 239) (see also Hornberger 2018; Quet 2021).

This article explores the trade of sex enhancers in The Avenues, a mixed residential and commercial area (including hotels) in the periphery of the Central Business District (CBD) in Harare, Zimbabwe. One of the hotels is the Bronte Hotel, a luxury establishment in The Avenues, a residential area where families of usually middle-class citizens live. It is in these streets of The Avenues that the sex trade and the trade in sex enhancers is concentrated. Here, sex enhancers are called “order” or *mapiritsi* (plural for *piritsi)*. *Piritsi* is a Shona word derived from the word pill. When I first arrived in streets of The Avenues, I was confident that I would find “fakes” as these spaces were unregulated and part of the so-called underworld or shadow economies. The issue of fake sex enhancers is also inextricably intertwined with discourses around efficacy. “There are no fake sex enhancers”, I was told when I posed the question to the traders. I asked if their products always worked effectively and satisfactorily. My assumption was that a fake product was one that claimed to do one thing, and yet did nothing or did something else. It was a very simplistic conceptualisation. I noticed that fakery was not a common subject of discussion among traders themselves. Indeed, my experience seems to echo that of David Mills and his co-authors, who argue that objects are not essentially seen as fake but certain practices and actors construct fakery around objects “leading to the raising of suspicion” (2021, 280). In this case, it was the state and the society at large that saw the streets as havens of fakery.

The selling of sex enhancers in urban Harare’s The Avenues district is done in open spaces in the streets. In the outskirts of the city, traders are found in the streets waiting for clients – most of whom will be driving. Some of these streets are parallel to and run across Fife Avenue. The whole surrounding area has come to be called The Avenues and, over the years, has become notorious as a red-light district (see Uledi and Kufakurinani 2020). The location of traders is no coincidence as sex enhancers feed from and into the sex trade dominant in the area. Around The Avenues area, during the day, small groups of traders, mostly men, can be seen in the streets, some touting different brands of sex enhancers and describing their efficacy to potential clients. If you slow down driving past the area, a score of traders (or in organised spots only one trader) either rush to your car with their merchandise or extend some signs of advertising their merchandise. Prices range from USD1 to USD5 for a product depending on a variety of factors.

From my research, I discovered that sex enhancers were divided into herbal and non-herbal products. These were sold in different forms which included pill forms (capsules and tablets), coffee powder, oils and liquids. Oils were usually smeared on the male organ while liquids were packaged in sachets and would be consumed before a sexual encounter. On average, these enhancers were to be taken between 15 and 45 min before sex for greater efficacy. In the non-herbal pills, the active ingredients were mostly sildenafil and tadalafil. The former is the active ingredient in Viagra and these products are generic drugs, coming from mostly China, India and Zambia.

## Navigating the Research Field

I spent over six months doing fieldwork and this involved spending time with traders, accompanying them to meet clients as well as conversations with friends and colleagues about sex enhancers. While The Avenues was my primary research site, I always felt that my field site was elastic and fluid. Every place I went was a potential source of information. Even social media offered interesting information, particularly on the advertising of sex enhancers.

I failed to interview traders a number of times before I managed to get Misheck^3^ to listen to me. It was relatively easy with Misheck because we happened to come from the same neighbourhood. He grew up in the neighbourhood where I now live, and we were able to reflect with nostalgia on several places we both knew. By the time I introduced my research, Misheck and I were now less of strangers to each other. Once I got the attention of Misheck and his trust, it became relatively easy to be introduced to more traders but it still took time before I could sit comfortably in their midst and share stories and laughter. I later understood their caution and at times aggression towards strangers who were not coming as clients. Drug busts from real and fake police were a real threat and they could not be too trusting.

Getting information from clients, who are largely male, was far more difficult. One cannot randomly approach people to talk about their sex life. This is because most aspects about sex are a taboo subject matter in Zimbabwe, as it is amongst many African cultures. People rarely discuss their sexual exploits especially with strangers, even more so if this is to discuss perceived challenges of erectile dysfunction. This posed challenges especially concerning accessing clients. As a way around this, I approached people in my network, colleagues, friends or relatives and, among these, two acknowledged that they did use sex enhancers and were willing to share their experiences.

The research was complicated by the Covid-19 pandemic, which restricted movement through nationwide lockdowns. The first lockdowns were total lockdowns in most parts of the world with conditions softening over time. In Zimbabwe, though conditions relaxed, movement remained restricted for a while. The first lockdown had the tightest restrictions with movement of non-essential services coming to a halt. From the second lockdown onwards, informal traders like those at Fife Avenues were able to negotiate their way around travel restrictions by using documents that designated them as essential services. During the second lockdown I stopped visiting the field site only to begin again when the restrictions were lessened. Even then, my visits were not frequent. During the period I could not visit or had to reduce my visits, I was told that business was relatively slow partly because of restricted movements. Night life was heavily reduced by the curfews and most traders who used to do night trading switched to day shifts while some tried alternative avenues of making money. Other traders extended their trade to online platforms such as WhatsApp where they could meet up with a wider pool of clients and sell their products. Misheck continued to come to his usual spot and so I entered into some agreement with him to post to me via WhatsApp his summary of his day’s experience. Though he was not consistent on this, I still managed to document some of his days’ experience which consisted of the traders’ engagement with the police and even some experiences with clients. He also sent me “transcripts” of their conversations on the traders’ WhatsApp group. Here, discussions were largely about the movement of the police, whom the traders dreaded and saw as their greatest threat. Apart from ethnographic observations and the entries from Misheck I also held a one-on-one interview with a female trader, Ellen.

As I worked on this research, I faced challenges engaging officials from the Ministry of Health and Child Welfare, MCAZ as well as formal pharmacists. I sought to understand their perspectives on sex enhancers sold in the streets. Unfortunately, there was a great deal of red tape trying to access these institutions and a general unwillingness to cooperate. The excuse I received from both the Ministry and MCAZ was Covid-19. They had suspended non-essential activities and general engagement with outsiders. I sent in my emails of requests for information, but received no response until right at the end when officials from MCAZ accepted to work with me and assigned someone who, unfortunately, was always travelling and difficult to reach. I eventually settled to work without these institutions for the moment. As for the pharmacists, I can only speculate about the reasons for their unwillingness to talk about these issues. It is possible that they may have suspected that I was an official from MCAZ trying to investigate their activities. I tried with three pharmacists and after failing to get their cooperation, I decided to leave them out.

The research makes use of both mainstream and social media and, on some occasions, I was able to get a glimpse of an insight into the role of the state from these. The issue of illicit drugs in general and sex enhancers has become a heated subject in the country and, over the years, it has attracted the attention of the public media. Newspaper articles include stories that are a result of investigative journalism and allow insights into what may otherwise be difficult to access as a researcher. Admittedly, investigative journalism is itself not without its own limitations (for more discussions on this see Atuire et al., 2021). Social media spaces such as Twitter handles are also quite fascinating as they offer insights from and perspectives by the general public. The availability of information of this nature, at a click, has made it possible to have a contextual appreciation of the trade and consumption of sex enhancers, especially as conceptualised by authorities and within official circles.

## Understanding and Interpreting the Efficacy of Sex Enhancers in Harare’s Streets

Sjaak van der Geest, Susan Reynolds Whyte and Anita Hardon have argued that pharmaceuticals are a social and cultural phenomenon with their own “life cycles” “from production, marketing, and prescription to distribution, purchasing, consumption, and finally their efficacy” (see also Whyte et al. 2003). They add that “each phase has its own particular context, actors, and transactions and is characterized by different sets of values and ideas” (van der Geest et al. 1996, 153). I build on this and understand efficacy as one of these stages of the drug’s life cycle. Drug efficacy as a point of departure allows us to explore multiple sites. First, it allows us to understand the anatomy of fake talk (talk about fakeness) around sex enhancers as it relates to perceived efficacy or lack thereof. Second, it allows us to explore the rationale of the state as it enacts laws against certain drugs. Third, this can be a useful window into understanding the perspectives of society (clients and non-clients) towards sex enhancers. I am also interested in the knowledge systems that traders have about the degrees of efficacy of various types of sex enhancers on the market and how these knowledge systems are acquired and passed through to fellow traders and clients. Efficacy as a point of departure also allows us to get insights into why clients buy sex enhancers and explain their preferred choices. Finally, and more specifically, the trade in sex enhancers in The Avenues area gives us a glimpse into discourses around masculinities and sexuality among Zimbabwean men. The traders in The Avenues sell largely sex enhancers for men and over 95 per cent of these traders are male.

Cori Hayden discusses a complexity of factors that shape drug efficacy, and these go beyond the drug itself “since the people who consume drugs are likely to have different metabolisms, and hence may process drugs at different rates, and may experience different side effects as well” (2012, 276). Indeed, a closer analysis would throw us into deeper and wider discourses around drug efficacy as something determined by such aspects as “individual metabolism, brand loyalty, and inactive chemical components used in drug delivery” (Hardon and Sanabria 2017, 121). My interest, therefore, developed around understanding how the efficacy of sex enhancers was imagined and construed, and what this meant for the trade in *mapiritsi*, as the traders called them. The trade itself has evolved over the years and has become elaborate and popular. It self-regulates, promoting certain drugs and shunning others depending on the dictates of the market which are usually determined by perceived efficacy of the drugs and their availability on the market. Drugs that grow unpopular simply lose the market. As Kristin Peterson observes, traders in the informal markets are rational actors (2021).

The research takes its cue from a few critical works. The seminal works of Whyte, van der Geest and Hardon in their *Social Lives of Medicines* (2003) and Arjun Appadurai’s influential earlier work *The Social Life of Things* (1986) draw us to the significance of exploring the materiality of objects and their life cycles as they interact with social and economic phenomena. I seek to demonstrate the life cycles of sex enhancers and how they too have enacted social and economic life cycles in an urban set up in Zimbabwe. Another critical work is Anita Hardon and Emilia Sanabria’s *Fluid Drugs* in which they explore “the pharmaceutical object” to understand “efficacy as a processual, relational, and situated event, as well as a pharmacological one” (2017, 118). Sex enhancers have generated not just talks around efficacy but have also shaped social and economic lives among Zimbabweans. Informal markets have emerged, and livelihoods been sustained through sex enhancers in a volatile economic environment. Because sexual prowess and the ability to satisfy a woman sexually is associated with masculinity, sex enhancers, in a way, have also been used by men to “reclaim” their perceived lost masculinity. Certain men are thus able to use sex enhancers to control their bodies, however, temporarily. This echoes observations about drugs in general as “powerful in that they offer users a means of control” (Whyte et al. 2003, 15). This control, they add, “can lead to being controlled. Drug dependence is the most obvious form of subjection” (2003, 15). This complex web of cause and effect is tied to the idea of assumed efficacy in sex enhancers.

In response to my question on the efficacy of sex enhancers, the traders were honest. “No, sex enhancers do not always work as expected, but this is not because they are fake. It is all about compatibility with one’s blood system.” This was an insightful response, which was echoed in the scholarship which also shifted attention from the drug to the consumer (van der Geest et al. 1996, 166). Misheck stressed that generally sex enhancers in capsule form remained in the system for longer. Tablets on the other hand worked immediately and stayed in the system for a shorter time. However, as my informant stressed, it all boiled to compatibility with one’s blood system. “*Zvese zvinozongoenderana neropa rako*/eventually, it is all about how far the drug is compatible with your blood system”, adding that there are cases where sex enhancers fail to deliver to expectation for one person, but do so for another.

When I went online, Big Man, one of the popular products in the streets, was said to “effectively increase adrenaline and renew sperm” and to be able “to achieve more than one ejaculation and more than one orgasm – allowing you to spend many more hours in bed”. The tonic was also said to have the capacity to be in one’s system “for up to 48 hours – keeping you ready for action”.^4^ On the box, Big Man has a small print written “pure natural herbal extract”. Africa Large Banana on the other hand had less of a digital footprint. One website only listed the effects of the tonic as follows: “increase the time of intercourse, better ejaculation control, increase volume of ejaculate, increase in thickness, increase in stamina, experience rock hard erections [and] increases sexual confidence”.^5^ There are several other products on the market all claiming to do the same thing. I managed to pick at list 15 empty boxes or shells of different type of products from the trading spots. The names of the enhancers like “Big Man”, “Africa Large Banana” “Manforce”, “Manking”, “7hours”, “Wild Horse”, “Rock Hard Weekend” invoked images of the efficacy of the products. From my conversations with traders, Big Man and Africa Large Banana seemed to top popularity because of their perceived limited side effects and their alleged ability to last longer and more effectively in the blood system. Both were also said to be herbal and, therefore not to contain active chemical ingredients. The names of the enhancers also construct a certain type of masculinity.

As a result of their perceived efficacy, drugs like Big Man attracted a higher price on the market compared to other pills. On average Big Man was sold at USD5 per pill but could go as low as USD2. The wholesale price was around USD1 a pill. The pricing of pills was, however, not just about perceived efficacy – though this seemed to play a very important role. I noticed that traders also tended to use the perceived class of a client to determine price. A client driving a fancy car and one coming on foot were likely to be quoted different prices of the same product. On some occasions, some pills could run out on the market for a while and thus induce shortage and subsequent demand leading to price hikes. I remember this happened with Big Man at some point. A friend of mine who introduced me to this trade and was a regular client used to tell me stories about Big Man and how it disappeared from the market for a while, causing a huge spike in its price. While in the field, a similar thing happened with Africa Large Banana. Amongst eight or so traders, only one had it in stock and he was making a large profit selling a packet of ten pills for USD15. Usually, they could sell for USD10 per packet. Misheck did not think this had anything to do with Africa Large Banana itself as a product and its efficacy, for it competed well with Big Man. For Misheck, this was more to do with the supplier. He was convinced the supplier deliberately reduced or even cut supply from the market to induce demand for the product

Even as traders acknowledged the side effects of some of their products, they insisted that the problem was not with the enhancers per se, but just an issue of incompatibility. Therefore, one had to keep trying different pills until one found what was compatible with one’s blood systems. Misheck told me that most of his regular clients would call him asking for specific brands of sex enhancers. At one time, a client called him and asked him to deliver Manking in the CBD. The client preferred Manking to other sex enhancers because, as Misheck explained to me, other enhancers had side effects such as headaches and breathing difficulties. Sometimes Misheck convinced loyal clients to try other products especially when he did not have the client’s preferred products on him.

The products in the streets were presented as effective and without problem. Their efficacy, or lack thereof, was not contingent on their pharmacology but on the bodies of the recipients. This was a very interesting perspective. This challenged the existence of fake sex enhancers. In fact, the general response to the question of whether fake sex enhancers existed or not was always negative. If the fake entered their market, it would not last. The market would simply shun the products. The traders were also keen on keeping a good name of their streets, it was their source of livelihood after all, and they would not want to destroy this by quick gains that would destroy the image of their trade. This confirms Peterson’s observation of the informal traders as rational actors (2021). I witnessed one occasion traders collectively protecting the image of their trade. On one of the days, about five traders rushed to a client’s car and, while there, each trader tried to out-compete the other and one of the traders made a remark to the client to the effect that he sold original products, while his competitors had fakes. When the client left, other traders did not take it lightly. The remark was really intended to be just a marketing gimmick because the trader also sold the very same product that he had called fake. But his colleagues felt he had just gone too far. He was destroying the image of their trade over a few immediate gains, he was told. Other traders reprimanded their colleague for sowing seeds of distrust which might eventually destroy their source of livelihood. The traders clearly understood the importance of keeping a good name in the streets as this incident demonstrated.

Even Big Man had a counterfeit, I was later told, but this was not quite a counterfeit. It was a generic substitute. My informant, Kundi, had initially used the term “fake” Big Man but withdrew it immediately indicating that it was not fake as it was still effective. He did not quite know where to place it. Mathieu Quet observes that fakeness is an unstable and fluid identity, “continuously built along social and technical interactions” (2021, 259). Kundi’s hesitancy in calling a different Big Man from the orginal “fake” seems to echo this observation by Quet. Kundi had been a trader for over five years and was a supplier of sex enhancers himself at some point before turning into a street trader. He told me that there were at least two types of Big Man in the streets, both blue in colour, but one was lighter and the other darker. Kundi believed one of these two was a generic substitute. He used the term “mimicry” suggesting that one was copying the other. One had to be an expert in the trade to notice the difference between the pills. In terms of efficacy, Kundi maintained that both were effective though the “original” Big Man, which was darker, was considered more effective. In fact, he would not sell the lighter Big Man capsules to a client who has been using Big Man for a while because their system would probably have got used to the original Big Man which was stronger. Therefore, the generic version would only be given to first-time users.

When MCAZ banned over-the-counter sale of sex enhancers in 2013, concerns were expressed around their side effects, safety, authenticity, and so-called addictive components. I wanted to find out the perceptions of traders and society given the existing label of sex enhancers, particularly those found in the streets. The traders indicated to me that there were two types of pills – one herbal and another non-herbal. The traders told me that herbal pills were made from natural products and were chemical-free. Because of this, I was told, they were less harmful or addictive. In fact, the herbal/non-herbal dichotomy was one of the marketing strategies used by the traders with the so-called herbal pills usually being more expensive. Before I began visiting Five Avenues shopping centre for my research, I had already come across adverts on social media (especially WhatsApp groups) where sex enhancers were being touted as natural products.

This kind of advertising is akin to what Douglas Haynes (2012) calls the “sell of masculinity”. The adverts prey on constructions of masculinity that emphasise sexual prowess amongst men. I had an interesting encounter surrounding the herbal/non-herbal dichotomy when I took a day off from field work to have my car fixed in the Kopje area just above Harare Street in the city. While waiting for my car to be fixed, a middle-aged woman came by selling some traditional herbs, some in powder form, some as roots and others as barks from trees. She told me they were sex enhancers. I took an interest and we began a discussion. I asked her how one would know her products were effective, and I referred to the capsules and tablets sold in the streets around Fife Avenues and how these have already made a name for themselves and, therefore, are able to attract clients. At that point, she then gave me a long lecture. First, she herself had at some point sold the pills but she abandoned the trade. The pills, she said, were addictive. One would not be able to function without them if one used them frequently. The so-called herbal pills, she added, were in fact not herbal at all and had chemicals in them. The good thing about her traditional products, she said, was that they actually recovered all lost manhood through the [over] use of sex pills. Her herbs, she boasted, not only restored but also cleansed the system bringing it to its original state. And for those who had never used the sex pills, the herbs were still good and without side effects as they were natural. With herbs, there would never be any such thing as an overdose, she explained. She was a good and tempting advertiser, I thought. But perhaps more important were these contradictions and contestations around presentation and conceptualisation of efficacy and of what was herbal or natural.

## Efficacy Beyond the Pharmacological and Medical Domains

In this section, I discuss the discourses around efficacy, perceived or real, of sex enhancers beyond the medical domain. Writing about Viagra, V. Eugene Boisaubin and Laurence McCullough underscored the impact of the tonic beyond the physiological broadly influencing “emotional, psychological, and behavioral components of the patient’s life and well-being” (2004, 740). Boisaubin and McCullough explain “sildenafil is distinctive in that it has a direct impact upon the sexual lives, health, and general well-being of others, often identifiable others” (2004, 740). As such, sex enhancers have the power to create “a distinctive therapeutic relationship not only for the person taking it but also for others, their sexual partners”. Sex enhancers are thus drugs whose impact goes beyond the male consumer and with a potential to positively shape intimacy between partners. However, in this discourse pleasure is often conceptualised in male-centric ways. It is within this context that sex enhancers have been presented as an avenue to absolve a masculinity in crisis induced by a dampening economy. Beyond the consumers of sex enhancers, traders also gain livelihood from their sales to survive economic challenges in the country. Efficacy therefore is not just about the consumer. It is also about the supplier/trader and this is why it has been said that medicines have their own social lives (van der Geest et al. 1996).

The power of sex enhancers to restore manhood is a leitmotif amongst traders and even in adverts that circulate on social media. When I talked to women colleagues and friends, one had a different perspective about sex enhancers. Unlike most of the women I had talked to, she did not condemn the use of sex enhancers. She argued that men in Zimbabwe were under a great deal of social and economic pressure, and this affected their sexual performance. Sex enhancers helped them regain their lost masculinity. She added that if these were herbal, they would be safe. More preferably, they can take natural products like roots and plants known to be aphrodisiacs. This had been said also by one of the traders who had argued that the economic crisis deepened a crisis of masculinity which in turn promoted the consumption of sex enhancers. Patriarchy puts men under pressure to provide, protect and procreate. When men fail to meet these cardinal Ps of patriarchy, a crisis emerges (see Kufakurinani 2015). The economic crisis compromised men’s ability to meet the expectations of society, hence leading to a crisis of masculinity. Sex enhancers were repeatedly touted by traders and even in the online adverts as a panacea to lost manhood. It was not just men who were concerned with their lost manhood, even women partners became concerned and, on some occasions, used unscrupulous ways to try and recover this. In my field work, I came across stories of women who surreptitiously approached the traders for pills to administer to their unsuspecting husbands.

The trade in sex enhancers is largely for economic survival. Traders told me that they do not have a choice but to participate in this illicit trade. The huge unemployment rate in the formal sector worsens the prospects of survival for traders outside this illicit trade. Misheck is himself a trained builder. There were also qualified tilers, university students, graduates and even qualified teachers who were plying the trade. The selling of sex enhancers in the urban streets of Harare, like several other vending activities in the city, became a source of livelihood mostly for the unemployed. When I asked Misheck about the profitability of his business. He told me he made an average USD15 to USD20 a day and this enabled him to survive together with his wife and child. He worked mostly from Monday to Friday and occasionally on Saturdays. In terms of his expenses, he needed an average of USD200 a month. This included transport to work, food, bills, insurance, and clothing. He had a place of his own which he built in Chitungwiza, but was struggling to complete the building project. He was also contributing to the welfare of his parents and his in-laws for whom he was now a breadwinner. Misheck did not have any other source of income outside the trade in sex enhancers.

## Pharmaco-Epistemologies: Knowing and Knowledge about Sex Enhancers in the Streets

How did traders and their clients come to know about sex enhancers and their efficacy? How was information shared and spread amongst traders and with clients? I use the term pharmaco-epistemologies to refer to the knowledge that both traders and clients have and the processes of knowing about sex enhancers. I asked Misheck about how he became involved with sex enhancers. Amongst the traders, knowledge was passed verbally from one trader to another. When Misheck was at a low point financially, his brother-in-law, the husband to his wife’s sister, invited him to Fife Avenue shopping centre and apprenticed him into the trade in sex enhancers. Misheck now had some three or so years’ experience in the trade and boasted of a couple of loyal regular clients. For Misheck the knowledge he had of different products was passed down to him by a fellow trader. When a product was new, the supplier would also share with traders his knowledge of the product, which in turn would be shared with clients. Such knowledge included the known positive effects of the pills, possible side effects, how the pills were expected to work and the amount of time they spent in the body.

The young male traders themselves experimented with the products. Kudzi, for example, extended an invitation to me to also experiment with the products:

You should try some of the stuffs you know. I can give you for free. I can get you one that is safe … you cannot research a product that you have never tried out. … Otherwise, how do you know if what we are telling you is true … . I have tried almost every product I sell (extract from field notes).

I asked which was the *safe* drug and Kudzi mentioned Big Man. Auto-experimentation was thus one way in which the traders came to know about the pills. In a chat I had with a female trader, she told me that she came to know about the effects of the products via her male counterparts who experimented with the products and had shared this first-hand information about their efficacy.

Knowledge about the efficacy of products was also acquired through client reviews. If a product received too many negative reviews from clients, the traders would then know that it was not a good product, and the product was likely to lose confidence among traders and the clients. Traders would share such reviews. Clients, on the other hand, relied largely on the advice of the traders. Twice I witnessed Misheck encouraging his loyal clients to try out a different product which he said had the same effects as the products that the clients had originally wanted but which Misheck did not have in stock. In their 2014 research on use of psychoactive prescription drugs (PPDs) by young people in South Sulawesi, Anita Hardon and Amelia Ihsan have shown how the efficacy of Somadril was shared by word-of-mouth amongst sex workers (2014). Clients too shared their information amongst themselves about products which were popular. On social media platforms, for example, participants could discuss different forms of sex enhancers, where they were found and what their expected efficacy is. The adverts, such as the ones I described above, were also sources of information for clients. The boxes of some sex enhancers can give information on how they were expected to work.

## Conclusion

The article explores how products that are banned by the state acquire their own agencies in times of economic crisis. I have shown how efficacy is a fluid, elastic and complex phenomenon when it comes to sex enhancers. For example, from the traders’ perspectives the point of departure to understanding efficacy was not the sex enhancers but their clients and their body systems. The discourses around efficacy are themselves intricately intertwined with the broader political economy of the country. A deteriorating economy engendered a crisis of masculinity and, among other factors, contributed towards increased trade and consumption in sex enhancers. As I demonstrate in the article, efficacy goes beyond the pharmacological and medical boundaries. The drugs, for example, have become a source of livelihoods for the traders. There are several ways by which knowledge about the efficacy of sex enhancers is passed amongst both clients and traders, creating communities of knowledge and perpetuating the social life of the drugs.

## Notes

1 The Fast Track Land Reform Programme was embarked on by the Mugabe regime, ostensibly to address colonial land injustices by redistributing land from white agrarian capital to landless Zimbabweans. In many ways, however, this is regarded as a political strategy aimed at saving the face of the regime whose popularity was dwindling. The country’s economy was severely shaken by this land reform exercise.

2 “Zimbabwe Bans Sex Perfomance Enhancers” n.d. https://3-mob.com/health/zimbabwe-bans-sex-perfomance-enhancers/

3 All names of traders used here are pseudonyms. Misheck is one of my two main informants. The other informant is Kundi.

4 https://www.thedailysale.shop/products/big-man-hard-erection-capsules, accessed 6 April 2022.

5 https://www.kamagra-namibia.com/index.php?route=product/product&product_id=42, accessed 6 April 2022.
